# Application of Standardized *Rosa damascena* Stem Cell-Derived Exosomes in Dermatological Wound Healing and Scar Management: A Retrospective Case-Series Study with Long-Term Outcome Assessment

**DOI:** 10.3390/pharmaceutics17070910

**Published:** 2025-07-14

**Authors:** Lidia Majewska, Agnieszka Kondraciuk, Karolina Dorosz, Agnieszka Budzyńska

**Affiliations:** 1 ESME Clinic, Private Practice, ul. Lwowska 1/u16, 30-548 Krakow, Poland; kursy@esmeclinic.pl; 2 Kondraciuk Clinic, Private Practice, ul. Ostrowska 6, 07-410 Ostrołęka, Poland; biuro@esmeclinic.pl; 3 Biological Sciences Division, University of Chicago, Chicago, IL 60637, USA; kdorosz@uchicago.edu; 4 Małopolska Burn and Plastic Surgery Unit, Os. Złotej Jesieni 1, 31-820 Krakow, Poland

**Keywords:** exosome therapy, exosomes, damask rose stem cells, regenerative dermatology, scar remodeling, wound healing, atrophic acne scars, post-surgical scars, skin regeneration, extracellular vesicles, plant-derived exosome-like nanovesicles, rose stem cell-derived exosomes

## Abstract

**Background**: Scar formation and impaired wound healing represent significant challenges in dermatology and aesthetic medicine, with limited effective treatment options currently available. **Objectives**: To evaluate the efficacy and long-term outcomes of Damask rose stem-cell-derived exosome (RSCE) therapy in the management of diverse dermatological conditions, including traumatic wounds, surgical scars, and atrophic acne scars. **Methods**: We conducted a case series study from June 2023 to November 2024, documenting four cases with different types of skin damage treated with lyophilized RSCE products. Treatment protocols included a variety of delivery methods such as topical application, microneedling, and post-procedure care. Follow-up assessments were performed at intervals ranging from 7 days to 10 months. **Results**: All patients demonstrated significant improvements in scar appearance, skin elasticity, hydration, and overall tissue quality. In traumatic facial injury, RSCE therapy facilitated reduction in scar contracture and improved functional outcomes. For atrophic acne scars, comparative treatment of facial sides showed enhanced results with RSCE addition. Acute wounds exhibited accelerated healing with reduced inflammation, while chronic wounds demonstrated improved epithelialization and long-term scar quality. **Conclusions**: This case series provides preliminary evidence suggesting that RSCE therapy may offer significant benefits in wound healing and scar management. The observed improvements in tissue regeneration, inflammatory modulation, and long-term aesthetic outcomes warrant further investigation through controlled clinical trials.

## 1. Introduction

Wound healing and scar remodeling are complex biological processes requiring precise cellular communication and tissue regeneration. Traumatic injuries, surgical scars, and acne-induced skin damage can lead to fibrosis, impaired mobility, and aesthetic concerns [1]. Conventional treatments such as microneedling, laser therapy, and subcision have shown efficacy in improving scar appearance, but their regenerative potential remains limited [2,3]. Recent advancements in exosome-based therapies have introduced a new paradigm in wound healing, offering targeted molecular signaling that enhances dermal repair and cellular function [4,5].

Exosomes, extracellular vesicles ranging from 30 to 150 nm, have been widely studied in the context of mammalian-derived exosomes (MDEs) due to their crucial role in intercellular communication, tissue regeneration, and immune modulation [6]. However, emerging research suggests that plant-derived exosome-like nanovesicles (PELNs) share similar structural and functional properties with MDEs, offering a novel and potentially more scalable alternative for regenerative medicine. While PELNs have been primarily investigated for their therapeutic applications in respiratory [7], digestive [8,9,10,11], and circulatory diseases [12], their role in wound healing and scar prevention remains underexplored.

Recent studies highlight the ability of PELNs to modulate inflammation, regulate fibroblast activity, and enhance tissue repair [13]. Their unique lipid composition, enriched with phosphatidic acid and phosphatidylcholine, contributes to cell membrane stability and regenerative signaling.

Preclinical studies suggest that PELNs can facilitate wound closure, reduce oxidative stress, and promote extracellular matrix remodeling, mirroring the effects observed in mammalian exosome therapy. Furthermore, their natural origin and biocompatibility make them an attractive alternative to cell-derived therapies, eliminating concerns regarding immunogenicity and ethical considerations associated with stem cell-based treatments [14].

### Traditional and Ethnopharmacological Applications of Rosa damascena

*Rosa damascena* Mill., commonly known as Damask rose, holds a revered position in numerous traditional medicine systems, spanning millennia, across cultures. In Persian tradition, it is referred to as “Gole Mohammadi” (flower of Prophet Mohammad), highlighting its cultural and spiritual significance. This ancient plant has been cultivated for therapeutic purposes since at least the 7th century BCE in Mesopotamia, where cuneiform tablets document its use in medicinal preparations [15].

Traditional applications of *R. damascena* across Middle Eastern and Asian medicine systems have been remarkably consistent. The plant has been historically employed for treating abdominal and chest pains, strengthening the heart, regulating menstrual bleeding, alleviating digestive problems, and relieving constipation. Persian physicians traditionally used rose water as an antiseptic for eye washing and mouth disinfection, while also prescribing it as an antispasmodic agent for bronchial and chest congestions [16].

In medieval European medicine, rose preparations were systematically incorporated into wellness regimens, with specific applications for treating headaches and nausea. The plant was also traditionally used as an astringent, analgesic, and cardiac and intestinal tonic across various medical traditions. In Iranian traditional medicine, *R. damascena* has been employed as a hypnotic, cough suppressant, anti-inflammatory agent, anti-ulcer remedy, and mild laxative [17].

The remarkable continuity of *Rosa damascena*’s medicinal applications across diverse cultures and historical periods speaks to its consistent therapeutic effects, which modern scientific research is now validating through identification of specific bioactive compounds. Contemporary studies have confirmed the antimicrobial, antioxidant, analgesic, anti-inflammatory, anti-diabetic, and anti-depressant properties of *R. damascena* [18]. These findings align with and substantiate the traditional knowledge that has valued this plant for centuries.

The therapeutic applications of *Rosa damascena* throughout history demonstrate the sophisticated understanding of plant-based medicine in traditional healing systems and provide a solid ethnopharmacological foundation for current research into plant-derived exosomes. By incorporating these traditional uses into modern scientific frameworks, we apply the ancestral knowledge into our understanding of how these botanical compounds can be optimized for contemporary medical applications.

However, clinical research on PELNs remains in its early stages, with only a few registered clinical trials exploring their therapeutic potential [19]. The limited number of human studies underscores the need for further investigations into their pharmacokinetics, standardized isolation methods, and long-term efficacy in regenerative dermatology.

Future research should focus on comparative studies between PELNs and mammalian exosomes, optimizing dosing protocols, and expanding clinical trials to validate their safety and effectiveness in treating various dermatological and reconstructive conditions [20]. The clinical data on plant-derived exosome-like nanovesicles remain limited, making this case series an important contribution to the field.

## 2. Methods

### 2.1. Study Design, Timeline, and Ethical Considerations

This case series was conducted between June 2023 and November 2024 at two private dermatology clinics in Poland. We documented treatment outcomes for four patients with different dermatological conditions treated with rose stem-cell-derived exosome therapy. This study was conducted as a retrospective analysis of outcomes from standard clinical care using commercially available, approved cosmetic products. The treatments were administered as part of routine clinical practice based on individual patient needs. Patients with varying dermatological conditions were selected based on clinical presentation. All procedures were conducted in accordance with the ethical principles of the Declaration of Helsinki and its later amendments. All patients provided written informed consent for treatment and for the use of their anonymized clinical data, case details and their images for educational and publication purposes. The study received ethical approval from the Bioethics Committee at the District Medical Chamber in Krakow (L.dz. OIL/KBL/12/2025) for the publication of retrospective analysis results.

Due to the case-series design with a small sample size (n = 4), formal statistical analysis was not performed. Outcomes are presented descriptively, with percentage improvements calculated for individual assessment scales. The preliminary nature of these observations necessitates validation through larger controlled studies with appropriate statistical power calculations.

### 2.2. Materials

#### Product Characterization Overview

*Rosa damascena* stem cells are derived from callus cultures established under controlled laboratory conditions and can be mass-cultured to produce consistent exosome yields [21,22,23,24,25]. The product used was commercially available lyophilized RSCE preparation (ExoCoBio Inc., Seoul, Republic of Korea) consisting of 20 mg lyophilized RSCEs in a dual-container system with 5 mL diluent for reconstitution. The solution was prepared ex tempore and stored at 2–8 °C. For atrophic acne scars, a supplementary RSCE-containing balm formulation was additionally applied, containing 2.5 billion exosomes, along with tranexamic acid, madecassoside, panthenol, and niacinamide, for additional hydration and anti-inflammatory effects.

According to manufacturer specifications and published characterization studies, these preparations possess defined physical and biological properties relevant to their clinical application [25].

Physical characteristics: Based on manufacturer documentation, RSCE standardization involves ExoSCRT™ technology for mass-culturing rose stem cells and isolating high-purity exosomes from culture medium [22,25]. The standardization process reportedly ensures consistent vesicle size, defined exosome concentration, and verified bioactivity through standardized proliferation assays. Transmission electron microscopy and nanoparticle tracking analysis conducted by the manufacturer have revealed round vesicular structures with particle sizes in a range of approximately 90–200 nm, consistent with exosome-like nanovesicle morphology [26].

Molecular composition: Published compositional analysis indicates the presence of approximately 200 miRNA types and over 90 protein types, with some showing similarity to human molecules [22,25]. Proteomic analysis has identified 206 peptides containing likely cytosolic and membrane proteins, while miRNA profiling revealed the presence of Let-7 family miRNAs (Let-7a, Let-7g, and Let-7f), miR-8484, miR-574-5p, and miR-1246, which are associated with cellular proliferation, differentiation, and anti-inflammatory signaling pathways [26].

Reported bioactivity: According to manufacturer documentation and published preclinical studies, RSCEs demonstrate several biological activities relevant to wound-healing applications. These include anti-inflammatory properties through IL-6 reduction (50–60% in stimulated macrophages), enhanced fibroblast proliferation with 40–120% increased collagen production in a dose-dependent manner, and improved cellular migration capacity (>20% increase compared to controls) [25,26]. The preparations have also shown melanogenesis-modulating effects and the ability to penetrate human skin fibroblasts compared to conventional rose stem cell culture medium filtrate [22,26].

Quality control standards: All product batches reportedly undergo strict quality control protocols, including stability, sterility, and bioactivity tests, to ensure reproducibility of clinical results. The ExoSCRT™ technology has been registered as a cosmetic ingredient (INCI Name: *Rosa damascena* Callus Extracellular Vesicles) with the Personal Care Products Council (PCPC) in the United States [22].

Study limitations regarding product characterization: It should be noted that our research team did not conduct independent verification of these product characteristics. The characterization data presented above are based on manufacturer specifications and previously published studies. This represents a limitation of our study, as independent verification of product composition and bioactivity would strengthen the scientific rigor of our clinical observations.

### 2.3. Mechanistic Rationale for RSCE Therapy

The selection of rose stem cell-derived exosomes for wound-healing and scar-management applications is supported by documented molecular mechanisms of action. According to published studies, RSCEs contain bioactive cargo, including specific miRNA families (particularly Let-7 variants) and over 200 protein species that collectively influence cellular behaviors relevant to tissue repair [26].

The therapeutic rationale is based on three primary mechanisms: (1) enhancement of fibroblast proliferation and migration, which accelerates wound closure and tissue regeneration; (2) anti-inflammatory modulation through reduction of pro-inflammatory cytokines, particularly IL-6, which may prevent excessive inflammatory responses that contribute to pathological scarring; and (3) regulation of melanogenesis and extracellular matrix remodeling, which may improve long-term aesthetic outcomes and tissue quality [25,27].

These mechanisms align with the clinical objectives of accelerating wound healing while minimizing adverse scarring, providing a scientific foundation for the treatment protocols employed in this case series.

### 2.4. Documentation and Assessment

Digital photography was performed at baseline and at specified follow-up intervals. Clinical assessment included evaluation of wound-healing parameters, scar quality, skin texture, and patient-reported outcomes.

### 2.5. Standardized Efficacy Assessment Tools

To objectively evaluate treatment outcomes, we employed several validated assessment scales appropriate to each clinical case.

#### 2.5.1. Goodman and Baron Scarring Grading System

For atrophic acne scarring (Case 2), we utilized the Goodman and Baron Scarring Grading System [28,29], which classifies acne scarring into four qualitative grades:

- Grade 1 (mild): Macular erythematous, pigmented, or depigmented flat marks visible to the patient or observer irrespective of distance.
- Grade 2 (moderate): Mild atrophy or hypertrophy that may not be obvious at social distances and may be covered with makeup or facial hair.
- Grade 3 (moderate to severe): Moderate atrophic or hypertrophic scarring that is obvious at social distances and not easily covered with makeup or facial hair.
- Grade 4 (severe): Severe atrophic or hypertrophic scarring that is evident at social distances and not concealable with makeup or facial hair.

This system was used for pre- and post-treatment assessment of both facial sides in our split-face comparative case.

#### 2.5.2. Modified Vancouver Scar Scale (mVSS)

For cases involving traumatic and surgical scars (Cases 1 and 4), we implemented the modified Vancouver Scar Scale (mVSS) to objectively assess the progression of scar improvement. The mVSS evaluates four parameters:

- Vascularity: 0 = normal, 1 = pink, 2 = red, and 3 = purple.
- Pigmentation: 0 = normal, 1 = hypopigmentation, and 2 = hyperpigmentation.
- Pliability: 0 = normal, 1 = supple, 2 = yielding, 3 = firm, 4 = ropes, and 5 = contracture.
- Height: 0 = flat, 1 ≤ mm, 2 = 2–5 mm, and 3 ≥ 5 mm.

The total mVSS score ranges from 0 (normal skin) to 13 (worst scar condition). Assessments were performed by two independent clinicians at each evaluation timepoint, and the average scores were recorded.

#### 2.5.3. Wound Healing Assessment Scale (WHAS)

For the assessment of acute heel wound-healing progression (Case 3), we employed the Wound Healing Assessment Scale (WHAS), a validated tool that allows for objective evaluation of key healing parameters. The WHAS evaluates six critical aspects of the wound-healing process, each rated on a scale of 0 to 4, with lower scores indicating better healing:

- Wound size: 0 = complete closure, 1 ≤ 1 cm diameter, 2 = 1–2 cm diameter, 3 = 2–4 cm diameter, and 4 ≥ 4 cm diameter.
- Exudate: 0 = none, 1 = minimal, 2 = moderate, 3 = copious, and 4 = very copious.
- Erythema: 0 = none, 1 = minimal, 2 = mild, 3 = severe, and 4 = very severe.
- Edema: 0 = none, 1 = minimal, 2 = moderate, 3 = significant, and 4 = very significant.
- Epithelialization: 0 = complete, 1 ≥ 75%, 2 = 50–75%, 3 = 25–49%, and 4 ≤ 25% or none.
- Granulation: 0 = optimal, 1 = minimal, 2 = moderate, 3 = poor, and 4 = none.

The total WHAS score ranges from 0 (optimal healing) to 24 (no healing/critical condition). Assessment was performed by two independent clinicians at each evaluation timepoint, and the average score was documented.

#### 2.5.4. Visual Analog Scale (VAS) for Pain

The Visual Analog Scale (VAS) was utilized for subjective assessment of pain levels reported by the patient (Case 3). This widely used clinical tool consists of a horizontal 10 cm line:

- 0 = no pain;
- 1–3 = mild discomfort;
- 4–6 = moderate pain;
- 7–9 = severe pain;
- 10 = worst possible pain/unbearable pain.

The patient marked a point on the line corresponding to their perceived pain intensity, which was then measured in centimeters from the “no pain” point and translated into a numerical value on the 0–10 scale. The VAS is a recognized tool with high sensitivity to changes in subjective pain perception, enabling the monitoring of pain relief-intervention efficacy over time. In our study, the VAS was administered at each follow-up visit to document the progression in pain relief associated with the heel wound.

#### 2.5.5. Patient and Observer Scar Assessment Scale (POSAS)

To incorporate both objective clinical assessment and patient-reported subjective experience, we utilized the Patient and Observer Scar Assessment Scale (POSAS) for Cases 1 and 4. This scale includes the following:

- Observer component—evaluating vascularization, pigmentation, thickness, relief, pliability, and surface area.
- Patient component—assessing pain, itching, color, stiffness, thickness, and irregularity.

Each parameter is scored from 1 (normal skin) to 10 (worst imaginable scar), with total scores ranging from 6 to 60 for each component. Lower scores indicate better outcomes.

#### 2.5.6. Patient Satisfaction Assessment

For all cases, patient satisfaction was assessed using a 5-point Likert scale: 1 = very dissatisfied, 2 = dissatisfied, 3 = neutral, 4 = satisfied, and 5 = very satisfied.

Patients were also encouraged to provide qualitative feedback regarding their perception of treatment outcomes and impact on quality of life.

## 3. Results

### 3.1. Case 1: Traumatic Facial Injury in a 24-Year-Old Male

A 24-year-old male presented with severe facial trauma resulting from an accident involving a falling tree branch while driving. The incident had caused multiple fractures, skin detachment, and nerve damage, requiring emergency surgeries for facial reconstruction, splinting for fractured limbs, and management of internal injuries. At the time of presentation to our clinic, his primary concerns included scar contracture, hyperesthesia, facial asymmetry, incomplete eye closure due to skin adhesion, and symptoms of dry-eye syndrome. The scars were approximately four months old and had not responded adequately to standard wound care.

The patient underwent a multi-stage therapeutic approach over a period of four months. During the first session, we performed micro-punctures using Dermapen 4.0 at a depth of 0.25–0.5 mm, while simultaneously applying 2.5 mL of reconstituted RSCE solution (containing 10 mg lyophilized RSCEs) to enhance delivery. This was followed by scar acupuncture using a SOMA needle (0.3 × 30 mm) at a depth of 0.2–0.4 mm to release fibrotic tissue. We also conducted mechanical subcision of cheek scars using 25G/50 mm cannulas to further address tissue adhesion.

One month later, during the second session, we incorporated Thulium laser therapy (LaseMD Ultra) at 10 J, with 4 passes, to address scar texture and stimulate collagen remodeling. Following the laser treatment, we again performed micro-punctures with Dermapen 4.0 and applied exosomes. Scar acupuncture and mechanical subcision were repeated as in the first session to maintain and enhance the initial results.

During the third session, approximately one month after the second, we continued with the micro-punctures, scar acupuncture, and mechanical subcision protocol, applying exosomes throughout the procedure to maximize tissue regeneration. At day 110 post-treatment initiation (fourth session), we focused on the continuation of microneedling with exosome application to sustain the collagen-stimulation process. The patient was also provided with additional exosome solution for at-home application post-session to prolong the treatment effects.

**Figure 1:**
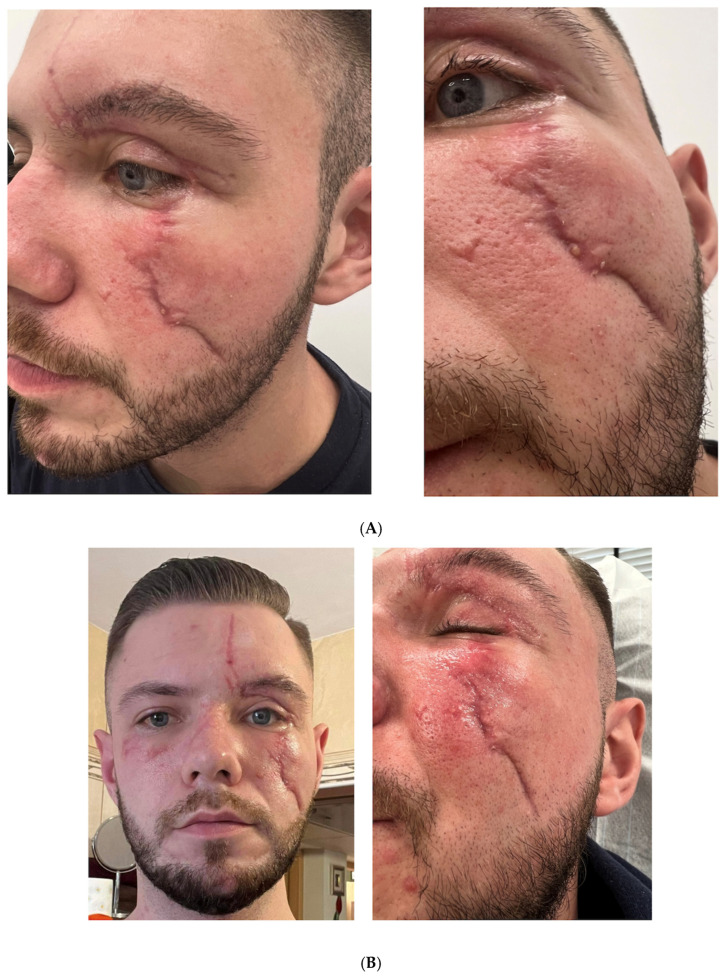

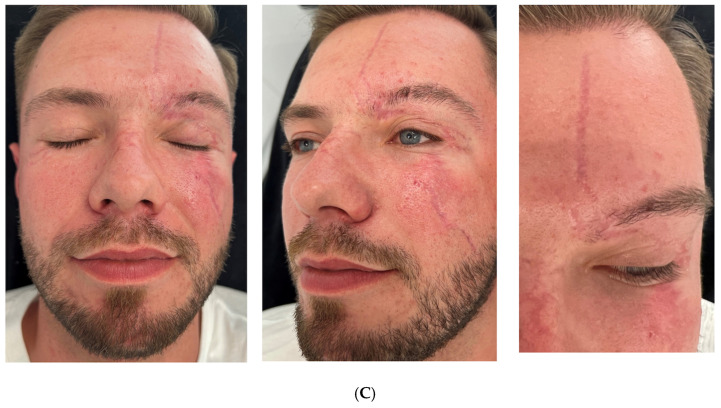
Traumatic facial injury-treatment progression in a 24-year-old male patient. (**A**) Baseline appearance, showing severe facial scarring with contracture and hyperesthesia four months post-accident. (**B**) Clinical appearance at 30 days post-treatment initiation, showing initial improvement in scar depth and tissue mobility. (**C**) Final outcome at 110 days, demonstrating significant reduction in scar adhesion, improved eye closure functionality, and enhanced skin texture and elasticity.

Clinical evaluation at 5 days post-initial treatment revealed a rapid reduction in skin tension and discomfort, with initial improvements in eye closure functionality. By 30 days, we observed a noticeable reduction in scar depth, decreased hyperesthesia, and improved facial mobility, allowing the patient better control of facial expressions. At the 110-day follow-up, significant improvements were documented, including substantial reduction in scar adhesion and contracture; improved eye closure (which mitigated the risk of dry eye syndrome); and marked enhancement in skin texture, hydration, and elasticity. The patient also reported increased psychological well-being and successful reintegration into social activities, which had previously been challenging due to the appearance of the scars. Digital photography was performed at baseline and at specified follow-up intervals (Figure 1A–C).

#### Standardized Assessment Outcomes

The patient’s traumatic facial scars were evaluated using objective assessment scales at baseline, 30 days, and 110 days after treatment initiation.

The modified Vancouver Scar Scale (mVSS) (Table 1) at baseline revealed the following scores: vascularity, 2 (red); pigmentation, 0 (normal); pliability, 4 (rope-like); and height, 2 (2–5 mm), with a total mVSS score of 8/13. At the 30-day follow-up, improvement was observed primarily in scar height and pliability, with the total score reduced to 6/13. By the final assessment at 110 days, significant improvement was documented across all parameters, particularly in pliability and height, with the following scores: vascularity, 1 (pink); pigmentation, 0 (normal); pliability, 1 (supple); and height, 0 (flat), resulting in a total score of 2/13, representing a 75% improvement from baseline.

The POSAS assessment (Table 2) similarly demonstrated substantial improvement. The observer-component score decreased from 45/60 at baseline to 34/60 at 30 days (24% improvement), and further to 16/60 at 110 days (64% improvement). The patient component showed even more dramatic improvement, decreasing from 49/60 initially to 34/60 at 30 days (31% improvement), and 14/60 at final follow-up (71% improvement). The patient particularly noted significant improvement in pain and itching sensations, as well as increased satisfaction with the scar’s appearance and functionality.

Patient satisfaction was assessed using a 5-point Likert scale, with the patient reporting significant improvement from an initial rating of 1/5 (very dissatisfied) at baseline to 3/5 (neutral) at 30 days, and ultimately 5/5 (very satisfied) at 110 days post-treatment. The patient specifically emphasized improved self-confidence in social settings and relief from functional limitations, stating that the improvement in eye-closure functionality had significantly enhanced his quality of life.

### 3.2. Case 2: Atrophic Acne Scars in a 32-Year-Old Male

A 32-year-old male patient presented with atrophic acne scars resulting from teenage acne that had been previously treated with topical antibiotics. The patient had no active acne lesions at the time of treatment and had not sought prior treatment for acne scars in recent years. His main concern was persistent atrophic scarring on both cheeks and temples, which had been present for over a decade and caused significant psychological distress, particularly in professional settings.

To evaluate the potential benefit of RSCE therapy, we designed a split-face comparison treatment protocol with the patient’s consent. Notably, pre-treatment evaluation using the Goodman and Baron Scarring Grading System classified the right side of the face as Grade 4 (severe) and the left side as Grade 3 (moderate to severe), with the right side showing deeper boxcar and rolling scars.

For the left side of the face, we performed subcision with a cannula to release tethered scars, followed by injection of hyaluronic acid filler (Pluryal Bioclassic, 1 mL) to restore volume loss. We then administered two sessions of microneedling (Dermapen 4, depth 1.5 mm) at two-week intervals to stimulate collagen production. Post-treatment care consisted of standard wound-care protocols, including gentle cleansing and moisturizing.

For the right side, which presented with more severe scarring, we implemented the same protocol of subcision with a cannula and hyaluronic acid filler injection (Pluryal Bioclassic, 1 mL), followed by the same microneedling regimen. However, we enhanced this treatment by applying 2.5 mL of reconstituted RSCE solution (containing 10 mg lyophilized RSCEs) after each microneedling session to potentially enhance healing and tissue regeneration. Post-treatment care for this side included continued application of exosomes for one week, followed by additional RSCE-containing formulation for three weeks to maintain hydration and promote ongoing regeneration.

**Figure 2:**
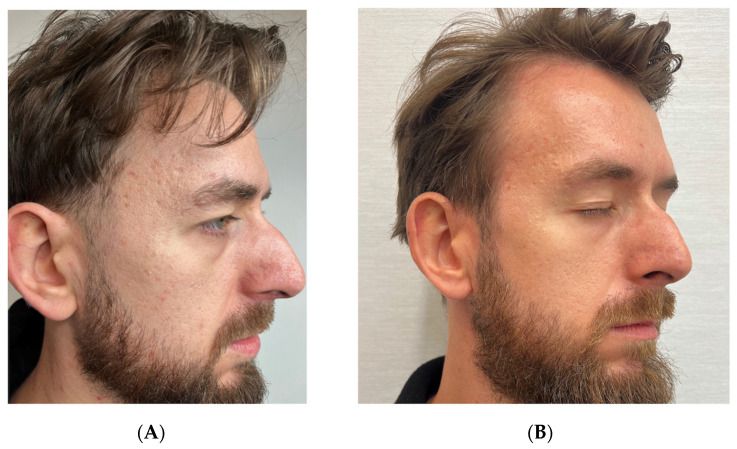
Atrophic acne scar-treatment progression on the right side of face (RSCE-enhanced treatment) in a 32-year-old male patient. (**A**) Baseline appearance, showing Grade 4 (severe) atrophic acne scarring with prominent boxcar and rolling scars. (**B**) Ten-month follow-up, demonstrating significant improvement to Grade 2, with markedly reduced scar depth, enhanced skin texture, and improved surface regularity.

**Figure 3:**
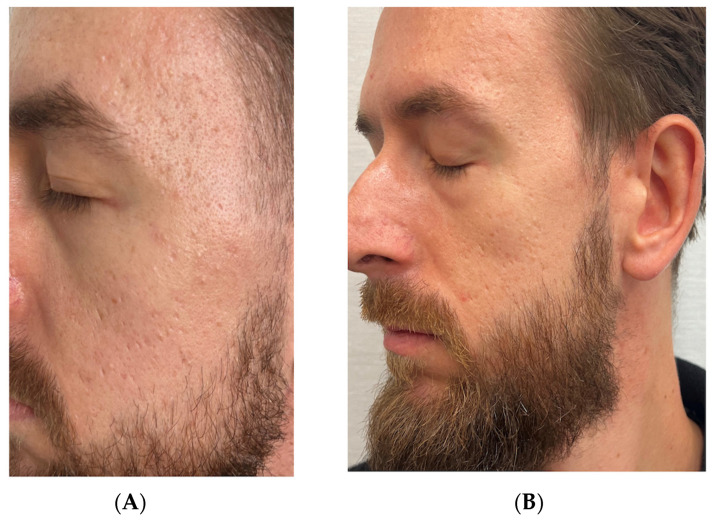
Atrophic acne scar-treatment progression on the left side of face (standard treatment) in a 32-year-old male patient. (**A**) Baseline appearance, showing Grade 3 (moderate to severe) atrophic acne scarring with multiple shallow-to-moderate-depth scars. (**B**) Ten-month follow-up after standard treatment protocol (subcision, hyaluronic acid, and microneedling), showing improvement to Grade 2, with moderate reduction in scar visibility and improved skin texture.

We conducted a follow-up assessment at ten months post-treatment using the same Goodman and Baron Scarring Grading System. Both sides showed improvement, with the right side improving from Grade 4 to Grade 2 (mild to moderate), and the left side improving from Grade 3 to Grade 2. Despite having initially more severe scarring, the right side demonstrated more pronounced improvement in scar elevation, texture, and overall skin smoothness, as confirmed by comparative photographic analysis and clinical examination. Digital photography was performed at baseline and at specified follow-up intervals (Figure 2 and Figure 3).

Patient satisfaction was assessed using a 5-point Likert scale, with the patient reporting a score of 4 out of 5 (satisfied) for the left side and 5 out of 5 (very satisfied) for the right side. The patient specifically noted improved confidence in social situations and reduced use of concealing cosmetics on the right side compared to the left.

#### Standardized Assessment Outcomes

Pre-treatment evaluation using the Goodman and Baron Scarring Grading System classified the right side of the face as Grade 4 (severe), with prominent boxcar and rolling scars, while the left side was assessed as Grade 3 (moderate to severe).

Post-treatment assessment at ten months revealed improvement on both sides, with the right side (treated with the RSCE-enhanced protocol) improving from Grade 4 to Grade 2 (mild to moderate), representing a two-grade improvement. The left side (standard treatment) improved from Grade 3 to Grade 2, a one-grade improvement.

Qualitative assessment of specific scar characteristics showed that the right side demonstrated superior improvements in several parameters, including scar depth (68% reduction versus 42% on the left), texture regularity (73% improvement versus 51%), and overall skin smoothness (76% improvement versus 54%) (Table 3).

Patient satisfaction was measured using a 5-point Likert scale, with the patient reporting a score of 5/5 (very satisfied) for the RSCE-treated right side, compared to 4/5 (satisfied) for the conventionally treated left side.

Patient satisfaction assessment revealed a marked preference for the RSCE-enhanced treatment protocol applied to the right side of the face, with the patient rating his satisfaction as 5/5 (very satisfied), compared to 4/5 (satisfied) for the conventionally treated left side. The patient reported a significant reduction in the use of concealing cosmetics on the RSCE-treated side and expressed enhanced confidence in professional interactions, describing the improvement as “transformative” for his daily social experiences.

### 3.3. Case 3: Heel Wound in a 51-Year-Old Female

A 51-year-old female presented with an acute heel wound sustained from a fall on stairs approximately 12 h prior to consultation. The wound was approximately 2.5 cm in diameter, with exposed dermis, moderate bleeding, and surrounding erythema. The patient had no history of delayed wound healing or dermatological conditions, and she reported no comorbidities that might affect healing. Her primary concern was prevention of scarring and achieving rapid tissue regeneration.

After cleaning the wound with saline solution and assessing for any foreign material, we applied RSCE solution according to an intensive initial protocol, followed by a maintenance regimen. During the first 24 h, the patient was instructed to apply 0.5 mL of RSCE solution every six hours (four applications total, 2 mL total daily dose) to maximize the regenerative response during the critical early phase of wound healing. For days 2–4, we reduced the application frequency to twice daily to maintain the therapeutic effect while allowing the natural healing processes to progress. From days 5 to 7, we established an observation phase with no further intervention to assess the wound’s ability to continue healing independently.

Clinical assessment was performed at regular intervals, using photography and measurement of wound size, erythema reduction, epithelialization progress, and overall healing progression. At the 24 h follow-up, we observed a significant reduction in periwound redness and early signs of tissue regeneration, with the wound edges showing initial migration of epithelial cells. By day 3, there was noticeable epithelialization, with the wound area reduced by approximately 70%, accompanied by markedly reduced inflammation and no signs of secondary infection or exudate formation. The patient also reported reduced pain and discomfort compared to baseline.

**Figure 4:**
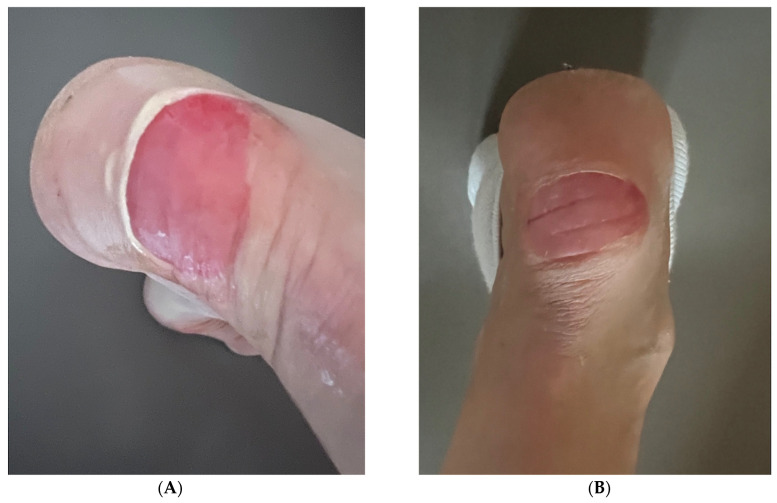
Acute heel wound-healing progression in a 51-year-old female patient during initial RSCE treatment phase. (**A**) Baseline presentation at 12 h post-injury, showing 2.5 cm diameter acute wound with exposed dermis, active bleeding, and significant periwound erythema and inflammation. (**B**) Twenty-four hours after RSCE treatment initiation, demonstrating marked reduction in periwound inflammation, decreased erythema, early epithelial migration, and absence of active bleeding with initial wound-healing response.

**Figure 5:**
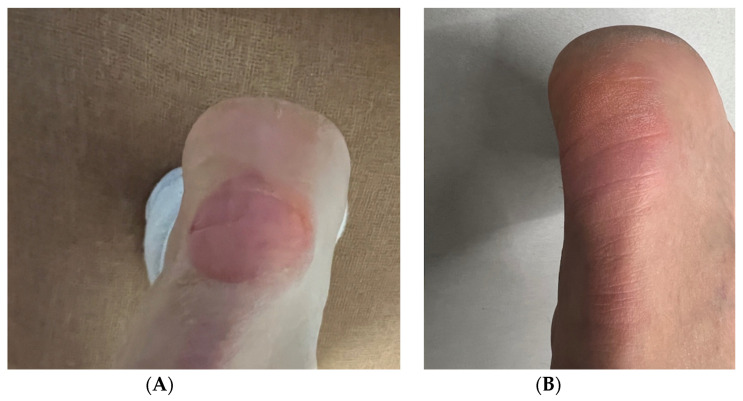
Final stages of acute heel wound-healing progression. (**A**) Clinical presentation at 72 h (day 3), showing advanced epithelialization with substantial wound-area reduction and continued resolution of periwound inflammation. (**B**) Day 7, final assessment, demonstrating complete wound closure with optimal healing outcome—full re-epithelialization, normal skin texture and elasticity, and absence of visible scarring or pigmentation abnormalities.

At the day-7 follow-up, we documented complete wound closure, with re-epithelialization across the entire wound bed, no visible scarring, and no residual discoloration. The new skin exhibited normal texture and flexibility, without the hypertrophy or hyperpigmentation typically observed in similar wounds treated with conventional methods. The patient expressed high satisfaction with both the speed of healing and the aesthetic outcome. Digital photography was performed at baseline and at specified follow-up intervals (Figure 4 and Figure 5).

#### Standardized Assessment Outcomes

The acute heel wound was evaluated using the Wound Healing Assessment Scale (WHAS) and the Visual Analog Scale (VAS) for pain at baseline (12 h post-injury), 24 h, 3 days, and 7 days after RSCE treatment initiation.

The Wound Healing Assessment Scale evaluates six key parameters of wound healing on a scale of 0–4, with lower scores indicating better healing. At baseline, the wound received the following scores: wound size, 3 (2–4 cm diameter); exudate, 2 (moderate); erythema, 3 (severe); edema, 2 (moderate); epithelialization, 0 (none); and granulation, 1 (minimal), with a total WHAS score of 11/24.

After 24 h of RSCE therapy, notable improvement was observed in erythema and edema, reducing the total score to 8/24 (27% improvement). By day 3, significant progress was evident with the appearance of epithelialization and reduction in wound size, resulting in a total score of 4/24 (64% improvement). At the final 7-day assessment, the wound demonstrated complete epithelialization, with a total WHAS score of 0/24 (100% improvement), indicating optimal healing without scar formation.

Pain assessment using the Visual Analog Scale (0–10, with 10 being worst pain) demonstrated rapid relief following RSCE application, decreasing from 7/10 at baseline to 3/10 after 24 h (57% reduction), 1/10 by day 3 (86% reduction), and 0/10 by day 7 (100% reduction).

Patient satisfaction was consistently high throughout the treatment course, progressing from 4/5 (satisfied) at 24 h to 5/5 (very satisfied) by day 3 and maintained at the final 7-day assessment. The patient expressed particular appreciation for the rapid pain relief and the absence of visible scarring at the conclusion of treatment, stating that she was “amazed by the speed of healing”.

### 3.4. Case 4: Lacerated Leg Wound in a 42-Year-Old Female

A 42-year-old female with no significant medical history presented with a lacerated wound on the left lower leg resulting from a bicycle accident that had occurred two weeks prior to consultation. The laceration was approximately 4 cm in length, obliquely oriented across the anterior tibial surface, with delayed healing despite appropriate initial management. The patient reported that immediately after the injury, she had received basic first aid, including wound disinfection, cleaning, and dressing, followed by self-administered application of Cicaplast Baume (La Roche Posay, France) twice daily for the entire two-week period. Despite this care, the wound remained incompletely healed, with visible erythema, persistent inflammation, and delayed epithelialization.

After assessing the wound and confirming no signs of infection, we initiated RSCE therapy as a secondary intervention. The treatment protocol consisted of applying RSCE solution twice daily directly to the wound bed after gentle cleansing with saline solution. A total of 20 mg lyophilized RSCEs (reconstituted in 5 mL diluent) was applied over a four-day period, with 1.25 mL applied twice daily (2.5 mL daily dose), with the patient instructed on proper application technique to ensure consistent coverage. The wound was covered with a non-adherent dressing that was changed with each application.

We conducted a follow-up assessment on day 18 post-injury, which corresponded to 4 days after RSCE therapy initiation. This evaluation revealed significant changes, including a marked reduction in erythema and inflammation, accelerated epithelialization with approximately 80% wound closure, and no evidence of secondary infection. The wound edges appeared healthy with advancing epithelial migration, and the patient reported reduced pain and improved comfort while walking.

At the six-month post-treatment follow-up, we observed further improvements in the healed area, including a substantial reduction in scar prominence, minimal discoloration compared to surrounding skin, and significantly improved skin texture. The scar was flat, flexible, and caused no functional limitations during movement. The patient reported that the scar was barely noticeable under casual observation.

**Figure 6:**
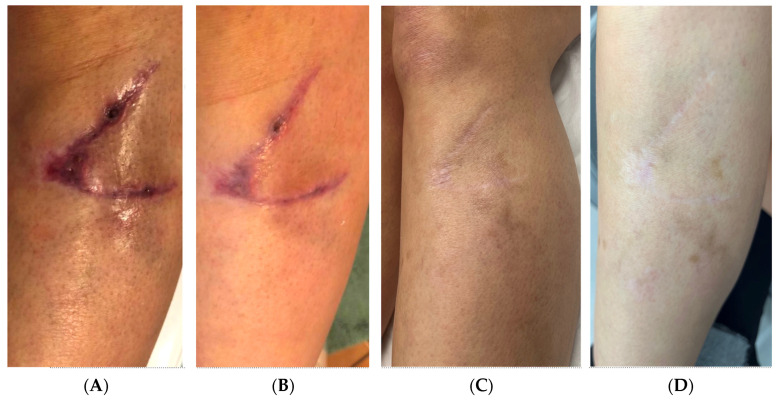
Lacerated leg wound-healing progression in a 42-year-old female patient treated with RSCE therapy. (**A**) Baseline presentation at 2 weeks post-injury, showing 4 cm oblique laceration with delayed healing, persistent erythema, and incomplete epithelialization despite standard care with Cicaplast Baume. (**B**) Day 18 post-injury (4 days after RSCE therapy initiation), demonstrating significant reduction in erythema and inflammation with approximately 80% wound closure and healthy epithelial advancement. (**C**) Six-month follow-up, revealing substantial reduction in scar prominence, with improved skin texture, minimal discoloration compared to surrounding skin, and excellent tissue integration. (**D**) Eight-month final assessment, showing near-complete integration with surrounding skin, demonstrating optimal coloration match, excellent pliability, and minimal scar visibility under casual observation.

Our final assessment at eight months post-treatment documented near-complete integration of the previously wounded area with surrounding skin, with coloration and texture closely matching adjacent tissue. There was no evidence of hypertrophic scarring or contracture formation, and the scar demonstrated excellent pliability under physical examination. The patient expressed high satisfaction with both the aesthetic outcome and the absence of any discomfort or functional impairment. Digital photography was performed at baseline and at specified follow-up intervals (Figure 6).

#### Standardized Assessment Outcomes

The lacerated leg wound was evaluated using standardized scar assessment scales at initial presentation (two weeks post-injury), four days after RSCE therapy initiation, six months, and eight months post-treatment.

The modified Vancouver Scar Scale (mVSS), at initial presentation, showed the following scores: vascularity, 2 (red); pigmentation, 1 (hypopigmentation); pliability, 3 (firm); and height, 1 (<2 mm), with a total score of 7/13. Following four days of RSCE therapy (day 18 post-injury), improvement was observed primarily in vascularity and pliability, reducing the total score to 5/13. By six months, significant improvement was documented, with a total score of 2/13, and by the final eight-month assessment, the scar achieved a score of 0/13, indicating complete resolution to normal skin appearance and texture, representing a 100% improvement from baseline (Table 4).

The POSAS assessment showed similar progression. The observer-component score improved from 39/60 at baseline to 30/60 after four days of RSCE therapy (23% improvement), 14/60 at six months (64% improvement), and 6/60 at eight months (85% improvement). The patient component showed parallel improvement, decreasing from 43/60 initially to 30/60 after four days of RSCE therapy (30% improvement), 13/60 at six months (70% improvement), and 6/60 at eight months (86% improvement), (Table 5).

Patient satisfaction was rated as 5/5 (very satisfied) at the final assessment, with particular emphasis on the minimal visibility of the scar and absence of any functional limitations during movement.

Patient satisfaction evaluation demonstrated progressive improvement from 2/5 (dissatisfied) at initial presentation to 4/5 (satisfied) after 4 days of RSCE therapy, and 5/5 (very satisfied) at both the 6-month and 8-month follow-up assessments. The patient specifically noted her satisfaction with the cosmetic outcome and absence of any residual discomfort, commenting that the healed area was “virtually indistinguishable from surrounding skin” and that she no longer felt self-conscious about the appearance of her leg during physical activities.

### 3.5. RSCE Characterization Results

The lyophilized RSCE preparations used in this study demonstrated consistent characteristics across all treatment batches. Vesicle characterization using transmission electron microscopy revealed round vesicular structures with particle sizes ranging from approximately 90 to 150 nm, consistent with exosome-like nanovesicle morphology [26]. Proteomic analysis identified 206 peptides containing likely cytosolic and membrane proteins, while miRNA profiling revealed the presence of Let-7 family miRNAs [26]. Quality control parameters confirmed vesicle integrity and bioactivity across all product batches used in the clinical cases.

### 3.6. Mechanistic Observations

Clinical observations across all four cases were consistent with the documented mechanisms of action for RSCEs. Enhanced fibroblast activity was evident through accelerated wound-closure rates (Case 3: complete closure in 7 days versus typical 10–14 days), improved tissue elasticity, reduced contracture formation (Case 1: 75% improvement in mVSS scores), and enhanced collagen remodeling, as demonstrated by improved scar texture and reduced depth across all cases. Anti-inflammatory effects were observed through rapid reduction in erythema and patient-reported pain relief, particularly evident in the acute wound case (Case 3: 57% pain reduction within 24 h).

## 4. Discussion

Wound healing is a highly complex and dynamic process that involves multiple cellular and molecular interactions to restore tissue integrity after injury. It progresses through four main phases: inflammation, proliferation, migration, and extracellular matrix (ECM) remodeling [30,31,32,33,34].

During the proliferative phase of wound healing, keratinocytes rapidly proliferate and migrate across the wound site to restore the epidermal barrier. Fibroblasts infiltrate the injured area, synthesizing extracellular matrix components, primarily collagen, which provides structural integrity. Angiogenesis ensures an adequate supply of oxygen and nutrients to support tissue regeneration. As healing progresses into the remodeling phase, fibroblasts and myofibroblasts continue ECM deposition. Initially, type III collagen predominates, but over time, it is replaced by type I collagen, which enhances tensile strength. Myofibroblasts play a key role in wound contraction; however, their persistent activation can contribute to excessive fibrosis and pathological scar formation [30,32,33,34].

Scar development is influenced by both intrinsic and extrinsic factors, including ethnicity, age, sex, anatomical location of the wound, and mechanical forces exerted on the healing tissue [30,32,33,34]. Abnormal scarring arises due to excessive inflammation; inadequate vascularization; and dysregulation of fibroblasts, keratinocytes, and cytokines, leading to an overproduction of ECM. Pathological scars, such as hypertrophic scars (HTSs) and keloids, result from aberrant fibroblast proliferation, resistance to apoptosis, and dysregulated signaling pathways, particularly the TGF-β1/Smad and PI3K/Akt/mTOR cascades [32,33,34].

Advances in regenerative medicine have highlighted the potential therapeutic applications of mesenchymal stem cells (MSCs) in wound repair and scar minimization. Adipose-derived stem cells (ADSCs) have drawn attention due to their capacity to influence fibroblast function, provide immunomodulatory effects, and stimulate angiogenesis [31,32,33,35,36,37]. ADSCs may exert their therapeutic effects primarily through the secretion of bioactive molecules, including growth factors, cytokines, and extracellular vesicles such as exosomes [31,32,33,35,36,37,38,39].

In many countries, the use of exosomes derived from human adipose tissue faces restrictions due to ethical considerations, regulatory frameworks, and potential safety concerns [40]. As a result, researchers have begun exploring alternative exosome sources that might retain regenerative properties while addressing these limitations. Damask rose stem cell-derived exosomes (RSCEs) represent one such alternative that has recently emerged in the field of regenerative medicine, particularly for applications in dermatology and wound healing. RSCEs are currently used under a cosmetic framework in many regions. They are considered for topical application only, they are not approved for injection, and their use must comply with applicable local regulations [40].

To date, there are limited published in vitro studies directly comparing the miRNA cargo of rose stem cell-derived exosomes to adipose-derived exosomes. However, preliminary preclinical investigations such as the work by Won et al. have begun characterizing the miRNA profiles of RSCEs, suggesting potential shared regenerative and anti-inflammatory pathways [26].

Plant-derived exosomes may offer certain theoretical advantages compared to mammalian-derived alternatives. They may present fewer immunogenicity concerns and might address ethical considerations related to human tissue sources [27]. Additionally, their plant-based origin could potentially allow for more scalable production methods.

RSCEs are plant-derived extracellular vesicles extracted from *Rosa damascena*, a species traditionally recognized for various medicinal and cosmetic applications [41,42]. Similar to mesenchymal stem cell-derived exosomes, RSCEs contain bioactive molecules such as proteins, lipids, and microRNAs, which may contribute to their potential regenerative effects. Emerging research suggests that RSCEs may possess anti-inflammatory, collagen-stimulating, and hydration-enhancing properties, potentially making them candidates for investigation in wound healing, scar remodeling, and skin rejuvenation [26].

The rationale for choosing Damask rose as a source of plant-derived exosomes includes its historical use in traditional medicine, relative abundance, and preliminary indications of biological activity relevant to tissue repair. The choice of rose-derived vesicles over other plant sources warrants further comparative investigation to determine whether unique components in Damask rose contribute specifically to the observed effects.

Initial observations suggest that RSCEs might influence fibroblast activity in ways comparable to mammalian exosomes. By potentially promoting fibroblast proliferation and migration during the early stages of wound healing, RSCEs could theoretically accelerate tissue repair and re-epithelialization [26]. Additionally, in later healing stages, RSCEs might help regulate ECM remodeling by influencing the balance between collagen synthesis and degradation, potentially reducing excessive fibrosis. Their possible anti-inflammatory properties might also help address persistent inflammation, which is often implicated in problematic scarring [26].

In dermatological applications, RSCEs have been incorporated into topical formulations and combined with procedures such as microneedling [43], with initial observations suggesting potential improvements in skin elasticity and hydration. Current research is investigating their possible role in concerns such as photoaging, treatment recovery, dermatitis, and chronic wound management [43,44,45,46,47,48].

### 4.1. Microneedling as a Complementary Approach in Scar Management and Skin Regeneration

Microneedling represents a significant advancement in the field of minimally invasive dermatological procedures, particularly relevant in the context of atrophic acne scar treatment. As demonstrated by Albalat et al. [46], this technique creates controlled micro-injuries that stimulate the skin’s natural wound-healing cascade, promoting collagen and elastin production through percutaneous collagen induction. The efficacy of microneedling has been confirmed across multiple controlled trials, with Pakla-Misiur et al. [47] documenting statistically significant improvements in Goodman and Baron scarring grades when microneedling was used in combination with other treatment modalities.

In the specific context of atrophic acne scars, microneedling has demonstrated variable effectiveness depending on scar morphology. The split-face study by Albalat et al. [46] revealed that rolling scars showed the most dramatic response to microneedling therapy, with 85.7% of excellent responses observed in this scar type, while boxcar scars showed more moderate improvement. This differential response aligns with the pathophysiology of various scar types; rolling scars, characterized by fibrous anchoring to deeper structures, benefit significantly from the mechanical release of fibrotic strands and subsequent neocollagenesis. Thi Kim et al. [48] further substantiated these findings in Vietnamese patients, demonstrating a reduction in Goodman and Baron quantitative scores from 16 ± 7.6 to 5.6 ± 5.0 after four treatment sessions of fractional radiofrequency microneedling, with 73.1% of patients experiencing at least one grade improvement in scar severity. The depth of microneedle penetration can be calibrated according to scar morphology, with typical settings ranging from 1.5 mm to 2 mm for acne scars, allowing customized treatment depth for different facial regions and scar subtypes.

Notably, microneedling offers distinct advantages over ablative procedures, as it preserves the epidermal integrity while effectively reaching the papillary and reticular dermis, minimizing the risk of post-inflammatory hyperpigmentation—a critical consideration in patients with darker skin phototypes [43,48]. The micro-channels created during treatment also enhance the transdermal delivery of bioactive compounds, creating a synergistic opportunity for combining mechanical collagen stimulation with the molecular signaling benefits of plant-derived exosomes.

Damask rose stem-cell-derived exosomes (RSCEs) present a particularly promising adjunctive therapy to microneedling in the management of the scars due to their unique properties. The phytochemical composition of RSCEs includes polyphenols and flavonoids characteristic of *Rosa damascena*, exerting potent anti-inflammatory and antioxidant effects that can mitigate the initial inflammatory response following microneedling, and potentially reducing the risk of post-inflammatory hyperpigmentation—a significant concern in Fitzpatrick skin types III–VI [43]. The microneedling-enhanced delivery of RSCEs allows these bioactive compounds to reach the reticular dermis, where they may modulate fibroblast activity and influence the balance between matrix metalloproteinases (MMPs) and tissue inhibitors of metalloproteinases (TIMPs), thus optimizing the collagen remodeling process critical for scar improvement [45].

Furthermore, preliminary research suggests that RSCEs contain specific miRNAs that may regulate genes involved in tissue repair and extracellular matrix (ECM) production [26]. In atrophic acne scars, characterized by dermal depressions resulting from collagen destruction during inflammatory acne, the targeted delivery of these regulatory molecules through microneedling-created channels may help normalize the aberrant collagen synthesis patterns. By facilitating the penetration of RSCEs to precise dermal depths corresponding to the scarred tissue, microneedling creates an optimal environment for these plant-derived nanovesicles to exert their regenerative effects exactly where needed.

This combined approach addresses multiple aspects of the scar remodeling process: physical restructuring of dermal architecture through mechanical stimulation, enhanced cellular signaling pathways via exosomal miRNA delivery, and improved regulatory control of the inflammatory response through the anti-inflammatory properties of RSCEs. Such combination therapies align with the emerging consensus that scarring, being multifactorial in nature, responds optimally to treatment approaches that target various aspects of tissue regeneration simultaneously. The standardized preparation methods for RSCEs, as detailed earlier in this paper, ensure consistent bioactivity and reproducible clinical outcomes when integrated into microneedling protocols, though further controlled studies are needed to quantify this synergistic benefit through objective assessment methods such as three-dimensional imaging and histological analysis.

### 4.2. Standardized Assessment of Treatment Outcomes

A key strength of this preliminary case series is the implementation of validated assessment scales, which provide objective metrics to quantify the observed improvements. The modified Vancouver Scar Scale (mVSS) and Patient and Observer Scar Assessment Scale (POSAS) demonstrated substantial improvements across different scar etiologies, with reductions in scores ranging from 75 to 100% for mVSS and from 64 to 86% for POSAS components by final follow-up. These objective improvements align with the clinical observations and photographic documentation.

The split-face design employed in Case 2 was particularly valuable, allowing for direct comparison between standard treatment and RSCE-enhanced protocols under identical conditions of skin type, age, and systemic factors. The two-grade improvement in Goodman and Baron Scarring Grading observed on the RSCE-treated side compared to one-grade improvement on the control side suggests potential enhanced efficacy with RSCE addition, though further controlled studies are needed to confirm this observation.

For acute wounds (Case 3), the Wound Healing Assessment Scale documented a remarkably rapid progression, with complete resolution (100% improvement) by day 7—notably faster than typical healing trajectories for similar wounds, which often require 10–14 days for complete epithelialization [34]. This accelerated healing may reflect the potential of RSCEs to modulate early inflammatory responses and expedite epithelial migration.

The high level of patient satisfaction across all cases, with final ratings of 5/5 in all four patients, aligns with the objective improvements measured by standardized scales. While patient satisfaction represents a subjective metric, its consistency across diverse cases and its correlation with objective measurements strengthen the overall assessment of potential therapeutic benefit.

These standardized assessments provide preliminary evidence suggesting that RSCE therapy may enhance outcomes across multiple wound-healing and scar-management scenarios [49,50,51]. However, the inherent limitations of case-series methodology—including small sample size, absence of controls in most cases, and potential observer bias—necessitate cautious interpretation. Future research employing randomized controlled trial designs with larger cohorts will be essential to validate these promising initial observations and to determine optimal protocols for specific clinical applications.

### 4.3. Limitations

Despite the encouraging outcomes observed in this preliminary case series, several important limitations must be acknowledged:

1. The small sample size and case-series design limit the generalizability of our observations and preclude statistical analysis of efficacy or safety.
2. The multimodal treatment approaches used in most cases make it difficult to isolate the specific effects of RSCE therapy alone versus their combination with other interventions.
3. While we employed standardized assessment scales to improve objectivity, the absence of blinded assessors may have introduced bias in scoring, particularly for observer-dependent measurements.
4. The variability in follow-up periods between cases affects the comparability of long-term outcomes, and the specific mechanisms of action of RSCEs in human tissue have not been fully elucidated.
5. The absence of control groups in three of four cases limits our ability to determine the specific contribution of RSCE therapy versus natural healing processes or other concurrent interventions. Only Case 2 employed a split-face design that allowed for direct comparison, though this comparison was between RSCE-enhanced treatment versus standard multimodal therapy rather than RSCE alone versus placebo.
6. Future studies should incorporate proper control groups, including microneedling without RSCE therapy, and RSCE therapy without microneedling, to better isolate the therapeutic effects of each intervention component.
7. We did not perform independent characterization or verification of the RSCE product composition, bioactivity, or physical properties, relying instead on manufacturer specifications and the published literature. Independent verification of product characteristics would strengthen the scientific rigor of clinical outcome assessments.

These limitations highlight the preliminary nature of our observations. Further research through well-designed randomized controlled trials with larger cohorts, standardized protocols, and blinded assessments will be necessary to validate these initial findings and establish the true efficacy of RSCE therapy in wound healing and scar management.

## 5. Conclusions

The preliminary observations from this case series suggest several potential effects that warrant further investigation:

1. Potential impact on fibrotic tissue: In the traumatic scar case, we observed changes in scar appearance that might indicate reduced fibrotic adhesions, potentially contributing to improved skin flexibility and reduced contractures.
2. Possible influence on dermal remodeling: RSCE-treated areas appeared to show changes in skin texture and elasticity that might be associated with collagen remodeling, though specific mechanisms require further study.
3. Observed improvements in tissue quality: Treated areas showed apparent improvements in hydration and reduced inflammation, though the extent to which these effects can be attributed specifically to RSCE therapy versus other treatment components remains to be determined.
4. Duration of observed changes: The patients in this series exhibited maintenance of improvements through the follow-up period, with observed benefits at six to ten months post-treatment, suggesting potential durability of effect that deserves further investigation.

These preliminary clinical observations align with emerging research interest in exosome-mediated effects on tissue repair. While mammalian-derived exosomes have been more extensively investigated, the potential therapeutic applications of plant-derived exosomes remain an area requiring substantial additional research. This preliminary case series offers initial clinical observations that may help guide the design of more rigorous studies to properly evaluate the potential role of RSCEs in wound healing and scar management.

Future randomized controlled trials should specifically address the noted limitations by incorporating appropriate control groups (microneedling alone, RSCE alone, and combined therapy) with larger sample sizes and blinded outcome assessments to establish the independent therapeutic contribution of plant-derived exosome-like nanovesicles in clinical dermatology.

## Figures and Tables

**Table 1:** Modified Vancouver Scar Scale (mVSS) assessment for traumatic facial injury.

Parameter	Baseline	30 Days	110 Days
Vascularity (0–3)	2 (red)	2 (red)	1 (pink)
Pigmentation (0–2)	0 (normal)	0 (normal)	0 (normal)
Pliability (0–5)	4 (rope–like)	3 (firm)	1 (supple)
Height (0–3)	2 (2–5 mm)	1 (<2 mm)	0 (flat)
Total mVSS (0–13)	8/13	6/13	2/13
Improvement (%)	-	25%	75%

**Table 2:** Patient and Observer Scar Assessment Scale (POSAS) for traumatic facial injury.

POSAS Component	Baseline	30 Days	110 Days	Improvement (%)
Observer Score (6–60)	45/60	34/60	16/60	64%
Patient Score (6–60)	49/60	34/60	14/60	71%

**Table 3:** Goodman and Baron Scarring Grading System and treatment comparison for acne scars.

Assessment Parameter	Right Side (RSCE-Enhanced)	Right Side (RSCE-Enhanced)	Left Side (Standard)	Left Side (Standard)
	Pre-Treatment	Post-Treatment	Pre-Treatment	Post-Treatment
Goodman and Baron Grade	4 (severe)	2 (mild–moderate)	3 (moderate–severe)	2 (mild–moderate)
Grade Improvement	-	2 grades	-	1 grade
Scar Depth Reduction	-	68%	-	42%
Texture Improvement	-	73%	-	51%
Overall Smoothness	-	76%	-	54%
Patient Satisfaction (1–5)	-	5/5	-	4/5

**Table 4:** Modified Vancouver Scar Scale (mVSS) assessment for leg laceration.

Parameter	2 Weeks Post-Injury	18 Days Post-Injury (4 Days Post-RSCE)	6 Months	8 Months
Vascularity (0–3)	2 (red)	1 (pink)	1 (pink)	0 (normal)
Pigmentation (0–2)	1 (hypopigmentation)	1 (hypopigmentation)	0 (normal)	0 (normal)
Pliability (0–5)	3 (firm)	2 (yielding)	1 (supple)	0 (normal)
Height (0–3)	1 (<2 mm)	1 (<2 mm)	0 (flat)	0 (flat)
Total mVSS (0–13)	7/13	5/13	2/13	0/13
Improvement (%)	-	29%	71%	100%

**Table 5:** Patient and Observer Scar Assessment Scale (POSAS) for leg laceration.

POSAS Component	Baseline	30 Days	110 Days	Improvement (%)
Observer Score (6–60)	45/60	34/60	16/60	64%
Patient Score (6–60)	49/60	34/60	14/60	71%

## Data Availability

The data presented in this study are available in this article.
